# A Case of Atraumatic Splenic Rupture Due to T-Cell/Histiocyte-Rich Large B-Cell Lymphoma and a Potential Role for Massive Transfusion Protocol

**DOI:** 10.1155/cris/4069182

**Published:** 2025-01-07

**Authors:** Sara Bohjanen, John P. Ratanawong, Mary Baumgartner, Bree Chandler, J. Carlos Manivel, Anthony T. Rezcallah

**Affiliations:** ^1^Medical School, University of Minnesota, 420 Delaware Street SE, Minneapolis, Minnesota 55455, USA; ^2^Department of Pathology, Minneapolis VA Health Care System, 1 Veterans Drive, Minneapolis, Minnesota 55417, USA; ^3^Department of General Surgery, Minneapolis VA Health Care System, 1 Veterans Drive, Minneapolis, Minnesota 55417, USA

## Abstract

Splenic rupture leads to massive hemorrhage and requires immediate surgical intervention. Splenic rupture results from trauma or from underlying disease processes. Lymphoma is a rare cause of atraumatic splenic rupture (ASR) with high mortality rates. We present a case of ASR due to T-cell/histiocyte-rich large B-cell lymphoma (THRLBCL) requiring splenectomy and large-volume transfusion. This case report highlights the necessity of prompt surgical intervention and massive transfusion for hemodynamically unstable ASR. This report also discusses massive transfusion protocol (MTP) and its limited use in nontraumatic surgical patients.

## 1. Introduction

Atraumatic splenic rupture (ASR) is a rare cause of hemoperitoneum associated with many underlying pathologies [1, 2]. Very few cases of spontaneous splenic rupture due to lymphoma have been published, and no case reports of splenic rupture due to T-cell/histiocyte-rich large B-cell lymphoma (THRLBCL) were found in our review of the literature. Splenic rupture carries a high risk of hemodynamic instability. When related to trauma, the diagnosis may be more straightforward. In cases of hemodynamic instability related to ASR, such as previously undiagnosed lymphoma, we are confronted with a diagnostic conundrum. Current management of hemodynamically unstable patients with splenic injury includes immediate surgical intervention, that is, splenectomy, with large-volume or massive blood product transfusions to achieve hemostasis and adequate fluid resuscitation [3]. Although large-volume transfusions are often required for acute splenic injuries, there is minimal guidance on massive transfusion in nontrauma patients [4]. Here, we discuss a rare case of ASR due to previously undetected THRLBCL requiring emergent surgical intervention with large-volume transfusion. We also discuss the potential role for massive transfusion protocols (MTPs) in nontrauma patients.

## 2. Case Presentation

The patient was a 61-year-old male transported to the emergency department (ED) via ambulance after being found somnolent during a well-visit check. En route to the hospital, the patient was diaphoretic and hypotensive to 80/40 mmHg. In the ambulance, he received a 3 L IV fluid bolus of isotonic solution with minimal improvement in his hemodynamics. He reported a sudden onset of epigastric abdominal pain with a sense of impending doom. Upon arrival at the ED, he was afebrile (36.3C), hypotensive (90/40 mmHg), and tachypneic (30 breaths/min). He had a pulse of 70 beats per minute and was noted to be on atenolol for hypertension, likely decreasing his ability to exhibit tachycardia. His oxygen saturation was 94% on room air. The patient was alert and conversant, and he reported a family history of ruptured abdominal aortic aneurysm (AAA) in his father.

On physical examination, his abdomen was distended and diffusely tender, but there was no palpable pulsatile mass, and his femoral pulses were equal bilaterally. The rest of the physical exam was normal. Bedside ultrasound could not adequately visualize the aorta. Further work-up revealed mild leukocytosis (12.9K/cmm), normocytic anemia (hemoglobin of 10.4 g/dL compared to a baseline of 15 g/dL, hematocrit of 32.8%), thrombocytopenia (109K/cmm), elevated troponin (0.123 ng/mL) with no acute EKG changes, elevated lactic acid (6.0 mmol/L) and INR of 1.34. Electrolytes were within normal limits.

**Figure 1:**
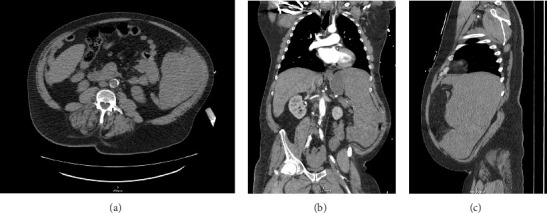
Computed tomography angiogram of the chest, abdomen, and pelvis in the (A) transverse, (B) coronal, and (C) sagittal planes, demonstrating enlarged spleen with large adjacent hematoma extending into the patient's pelvis.

A CT angiogram of the chest, abdomen, and pelvis with and without contrast was performed to rule out AAA (Figure 1). Initial imaging showed no evidence of a ruptured aortic aneurysm but rather an enlarged spleen (~20 cm along the craniocaudal axis) with an adjacent hematoma extending into the pelvis. Enlarged right axillary lymph nodes measuring up to 5 cm and multiple poorly attenuated hepatic lesions were also observed.

Given the patient's hemodynamic instability and concern for splenic rupture, the ED staff consulted general surgery. The general surgery team promptly responded by going to the ED and attending to the patient at his bedside. The abdominal findings were corroborated, and the CT was reviewed in the patient's room with him. The patient provided emergency consent for exploratory laparotomy, and he was immediately transported to the operating room by the surgery attending and ED staff.

He underwent an emergent exploratory laparotomy with splenectomy. Intraoperatively, there was extensive hemorrhage and clotted blood throughout the left upper abdominal quadrant. The spleen was unrecognizable and hemorrhaging profusely. Temporary hemostasis was achieved by manual finger compression of the vessels within the splenic hilum until the anesthesia team could stabilize his blood pressure with IV fluids and blood products. The splenic artery and vein were ligated and divided, as were the short gastric vessels, and the spleen was removed. A thorough inspection of the remaining abdominal cavity revealed no gross injuries to surrounding structures. The estimated blood loss was 11 L, and the patient received 12 units of blood (5 L with cell saver), 6 units of fresh frozen plasma, 4 units of platelets, and 4.5 L crystalloid solution intraoperatively. He did not require administration of cardiac pressors during or after the procedure. His postoperative hemoglobin was 14.7 g/dL, platelet count was 160K/cmm, and INR was 1.22.

**Figure 2:**
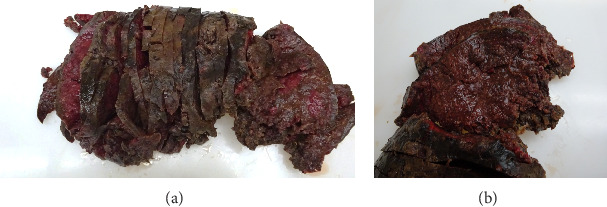
Photographs of the enlarged spleen and ruptured capsule (23.5 × 13.5 × 8.2 cm; 1.27 kg). (A) Sectioning revealed friable, soft-brown parenchyma and prominent white pulp. (B) Enhanced view resembling the intraoperative appearance of the spleen.

**Figure 3:**
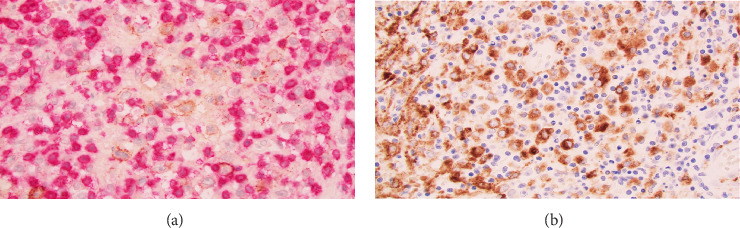
Microscopic histopathology of the splenic tissue. (A) Immunoperoxidase cocktail stain for CD3 and CD20 demonstrates numerous benign T cells (red) and few neoplastic B cells (brown). (B) Large numbers of reactive histiocytes stain positive for CD68.

Macroscopic examination of the pathology specimens demonstrated that the splenic capsule was diffusely disrupted, the splenic tissue was friable (Figure 2), and there were two lymph nodes within the hilar soft tissue. Histologically, the splenic hilar lymph nodes and splenic tissue demonstrated an extensive granulomatous reaction with multiple areas of dirty necrosis and areas with prominent hemophagocytic activity. Immunohistochemistry confirmed the presence of malignant B cells (positive for CD19, CD20, CD45, CD79a, PAX5, BCL2, BCL6, MUM-1, EMA, and Ki-67 and negative for CD3, CD15, CD30, CD68, ALK; also, Epstein–Barr virus-negative by chromogenic in situ hybridization). The scattered large B cells were in a rich background of histiocytes and T cells (Figure 3). Cytochemical stains for fungi, mycobacteria, and bacteria (GMS, AFB, Gram, Warthin-Starry) were negative. The morphology and immunohistochemical stains were consistent with a diagnosis of THRLBCL.

The patient's postoperative hospital course was complicated by pneumonia and a pancreatic leak requiring drain placement and cystogastrostomy with an AXIOS stent. The patient was eventually discharged on day 36 with oncologic follow-up. A subsequent PET scan demonstrated stage IVb lymphoma. The plan was for the patient to undergo a bone marrow biopsy, initiate rituximab, and later begin the R-CHOP chemotherapy regimen. However, the patient elected for hospice care and died within 4 months of his initial presentation. Although the patient decided against further treatment, the life-saving operation that he received allowed him to spend several precious months with his family, for which he was grateful.

## 3. Discussion

The presentation and evaluation of this patient suggested hemodynamic instability from internal hemorrhage. Ruptured AAA was initially suspected, but there are many causes of atraumatic hemoperitoneum. These causes can be classified as gynecologic, hepatic, splenic, vascular, and coagulopathic [1]. The most common etiologies are ruptured ovarian cysts, postprocedure hemorrhage, or bleeding mass [5]. The clinical presentation of atraumatic hemoperitoneum is often nonspecific, and diagnosis usually requires radiologic imaging [6].

CT imaging revealed findings suggestive of ASR in our patient. ASR is extremely rare and is most often caused by infections, such as malaria, Epstein–Barr virus, or cytomegalovirus [1, 2]. Additional etiologies include malignancy, amyloidosis, vasculitis, iatrogenic causes, and many others [2]. In our case, ASR was the first presenting sign of his THRLBCL, a rare and aggressive type of diffuse large B-cell lymphoma [7–9]. The overall mortality rate after ASR is 12.2%, and patients with neoplastic etiologies have significantly higher mortality [10].

Given his hemodynamic instability and evidence of splenic injury, immediate splenectomy was indicated [3]. With extensive splenic injury or trauma, patients often require extensive volume repletion and transfusion of packed red blood cells (RBCs), platelets, and clotting factors to prevent coagulopathy [3]. Massive transfusion is commonly defined as the transfusion of at least 10 or more units of packed RBC in the first 24 h of hospitalization [4]. In trauma patients, higher platelet:RBC ratios during massive transfusion are associated with decreased mortality [11]. MTPs have been developed by institutions to increase the utilization of plasma and platelets and to facilitate the delivery of blood products to hemodynamically unstable, hemorrhaging patients [4, 12]. MTP implementation for trauma patients is associated with decreased mortality [13–15].

Although our patient received a massive transfusion, an MTP was not activated. Often, nontraumatic patients do not have MTP activation for their hemorrhage. Most of the literature on MTP is focused on trauma patients, and there is limited guidance for nontrauma patients [4]. In a meta-analysis of four studies, there was no statistically significant effect of implementing an MTP on mortality rates for nontrauma patients [4]. However, the authors postulate that this nonsignificant effect, particularly when compared to the strong effect in trauma patients, may be due to a lack of statistical power, delayed activation from unclear criteria in nontrauma patients, and the increased age and medical comorbidities of nontrauma patients [4].

For our patient, he was successfully stabilized without implementing an MTP but required a large amount of blood products. He received care at a large tertiary care hospital, so there may have been fewer logistical constraints for transfusion compared to other hospitals. MTP facilitates the processing of blood products and is associated with faster delivery [16], which may be useful for medical systems with fewer resources. Additional data are needed to further elucidate the role of MTP for nontrauma patients, including whether it is beneficial for ASR and clinical presentations similar to our patient.

## Data Availability

The data supporting the conclusions of this case report are found within the article and by consulting the works cited.
